# Zebrafish xenografts in breast cancer research

**DOI:** 10.3389/fimmu.2025.1540610

**Published:** 2025-07-10

**Authors:** Xingyan Rong, Han Chen, Xiyuan Guo, Xiaoke Sun, Lin Li, Yingchun Ye, Chenwen Li, Siji Nian, Chengbi Liang, Qing Yuan

**Affiliations:** ^1^ Public Center of Experimental Technology, The School of Basic Medical Sciences, Southwest Medical University, Luzhou, Sichuan, China; ^2^ School of Stomatology, Southwest Medical University, Luzhou, Sichuan, China; ^3^ Institute of Cardiovascular Research, Southwest Medical University, Luzhou, Sichuan, China; ^4^ Component Department, Luzhou Central Blood Station, Luzhou, Sichuan, China

**Keywords:** zebrafish xenograft model, breast cancer, invasion, metastasis, angiogenesis, drug screening

## Abstract

Breast cancer (BC) tops the list of all malignancies diagnosed in women worldwide, with many patients diagnosed only at the metastatic stage. Current therapeutic paradigms integrating early detection modalities and multimodal treatment strategies have improved outcomes, yet persistent challenges in managing advanced/metastatic cases result in suboptimal 5-year survival rates. Therefore, it is imperative to develop novel therapeutic strategies for BC. Zebrafish breast cancer models have received great attention in this regard, and this review highlights recent advances in BC research involving these zebrafish models. *In vivo* research using zebrafish models is becoming increasingly valuable for studying BC invasion and metastasis, tumor angiogenesis, and screening for novel therapeutic molecules. These studies have provided insights into the molecular mechanisms of BC, potential drug targets and their efficacy and toxicity, and the application of zebrafish in personalized medicine research. Against this background, this review provides a systematic analysis of the recent advances in zebrafish BC model research regarding brain metastasis, bone metastasis, tumor angiogenesis, and drug screening. The review also critically evaluates the strengths and limitations of the zebrafish model organism, while delineating the future research directions in this field.

## 1 Introduction

Breast cancer (BC) has replaced lung cancer as the most frequently diagnosed cancer in women globally. The International Agency for Research on Cancer (IARC) estimates that approximately 19.3 million new cancer cases and 9.7 million cancer-related deaths occurred globally in 2022 (1). Metastatic progression remains a primary contributor to mortality in breast cancer patients, frequently demonstrating resistance to conventional therapies. The American Cancer Society and the National Cancer Institute reported 313,510 new breast cancer in 2024, with approximately 42,780 resulting in mortality (2). Triple-negative breast cancer (TNBC), characterized by the absence of estrogen receptor (ER), progesterone receptor (PR), and human epidermal growth factor receptor 2 (HER2) expression, represents the most aggressive breast cancer subtype. Accounting for 10–20% of all breast cancer cases (3), TNBC exhibits aggressive features including tumor heterogeneity, rapid metastasis to distant organs (particularly the brain, lungs, and bone) (4), and a high recurrence rate. Unlike hormone receptor-positive breast cancer (HR+ BC)—the most common subtype (5)—TNBC does not respond to endocrine or HER2-targeted therapies, and its treatment primarily relies on chemotherapy (6). In contrast, HR+ BC typically progresses more slowly, driven by hormonal signaling pathways (5), and benefits from well-established targeted treatment options such as endocrine therapies (7). This biological divergence extends to preclinical modeling: The scarcity of clear therapeutic targets in TNBC necessitates high-throughput drug screening using zebrafish models (8), whereas HR+ BC often fails to develop a typical phenotype in zebrafish. Furthermore, modeling HR+ BC resistance mechanisms (e.g., endocrine therapy resistance)—which involve significantly more complex host-tumor interactions (9)—is inherently limited in zebrafish systems. These characteristics pose significant therapeutic challenges. Zebrafish models have emerged as valuable tools for BC (especially TNBC) research, enabling the identification of genes underlying invasive metastasis and tumor angiogenesis, while facilitating the development of targeted therapies.

Over the past two decades, zebrafish (Danio rerio) has developed into an indispensable model organism for cancer research. Through the construction of various transgenic lines, its application value has been significantly increased, especially in the real-time monitoring of tumor angiogenesis and the study of tumor immune microenvironment, which demonstrates its unique advantages. The zebrafish embryonic vascular system is highly conserved evolutionarily, and its functional circulatory system can be constructed within 24 hours after fertilization (10). The optically transparent nature of embryonic tissues provides an ideal window for dynamic observation of developmental biological processes and pathological changes. By fluorescently labeling vascular endothelial cells, researchers can observe the early stages of tumorigenesis with high spatial and temporal resolution, tracking the behavior of neovascularization (10). Conversely, a dual-fluorescent-labeling system for tumor cells and host cells enables precise dynamic analysis at the single-cell level during metastasis (11). In addition, the high fecundity of zebrafish, the *in vitro* developmental characteristics of the embryo (10), and the tiny size of the zebrafish provide significant convenience for experimental manipulation. Together, these features enable the model to maintain a highly developmentally synchronized population of embryos in a limited space. Based on its cost-effectiveness, developmental homogeneity and miniaturization, the zebrafish system has become an ideal platform for high-throughput screening for potency assessment and toxicity testing of anticancer drugs. In this review, the multidimensional application of the zebrafish model in BC research will be systematically elaborated, focusing on its research progress in the areas of metastasis and invasion mechanism, tumor angiogenesis regulation and preclinical drug screening.

## 2 Establishment of zebrafish breast cancer model

Conventionally, genetically engineered murine (GEM) models and human cancer cell-derived xenograft models in immunodeficient strains (e.g., NOD-SCID) (12) have served as gold-standard platforms for preclinical evaluation of anticancer therapy safety and efficacy. Nevertheless, murine models present limitations (12) including prolonged experimental timelines and technical constraints that hinder high-throughput drug screening and comprehensive toxicity profiling. The zebrafish model has consequently emerged as a transformative platform in cancer research, demonstrating unique capabilities in revolutionizing precision oncology and advancing personalized medicine approaches.

Zebrafish cancer models are constructed using three main strategies: genetic engineering modification, chemically induced carcinogenesis and xenograft models. In the genetic engineering strategy, researchers have induced tumorigenesis by targeting and integrating oncogenic gene expression vectors or using targeted knockdown of tumor suppressor genes mediated by the CRISPR/Cas9 system (13). However, this model system has translational medicine limitations, mainly reflected in the lack of BRCA1 direct homologous gene in the zebrafish genome and only 22% amino acid sequence identity between its brac2 gene and human BRCA2 (14). To break through this limitation, the research team developed a morpholino oligonucleotide-mediated BRCA2 gene silencing model and a CRISPR knockout line, and these model systems provide key experimental evidence for resolving the DNA damage repair mechanism during breast cancer development (15). Chemically induced carcinogenesis allows for the establishment of mechanistically well-defined tumor models in multiple organ systems such as the liver, pancreas, gastrointestinal tract, epidermis, musculoskeletal system, vasculature, and testis through the exposure of specific carcinogens (16–20). The lack of mammary organs to induce breast cancer in zebrafish can be compensated for by xenotransplantation.

**Figure 1:**
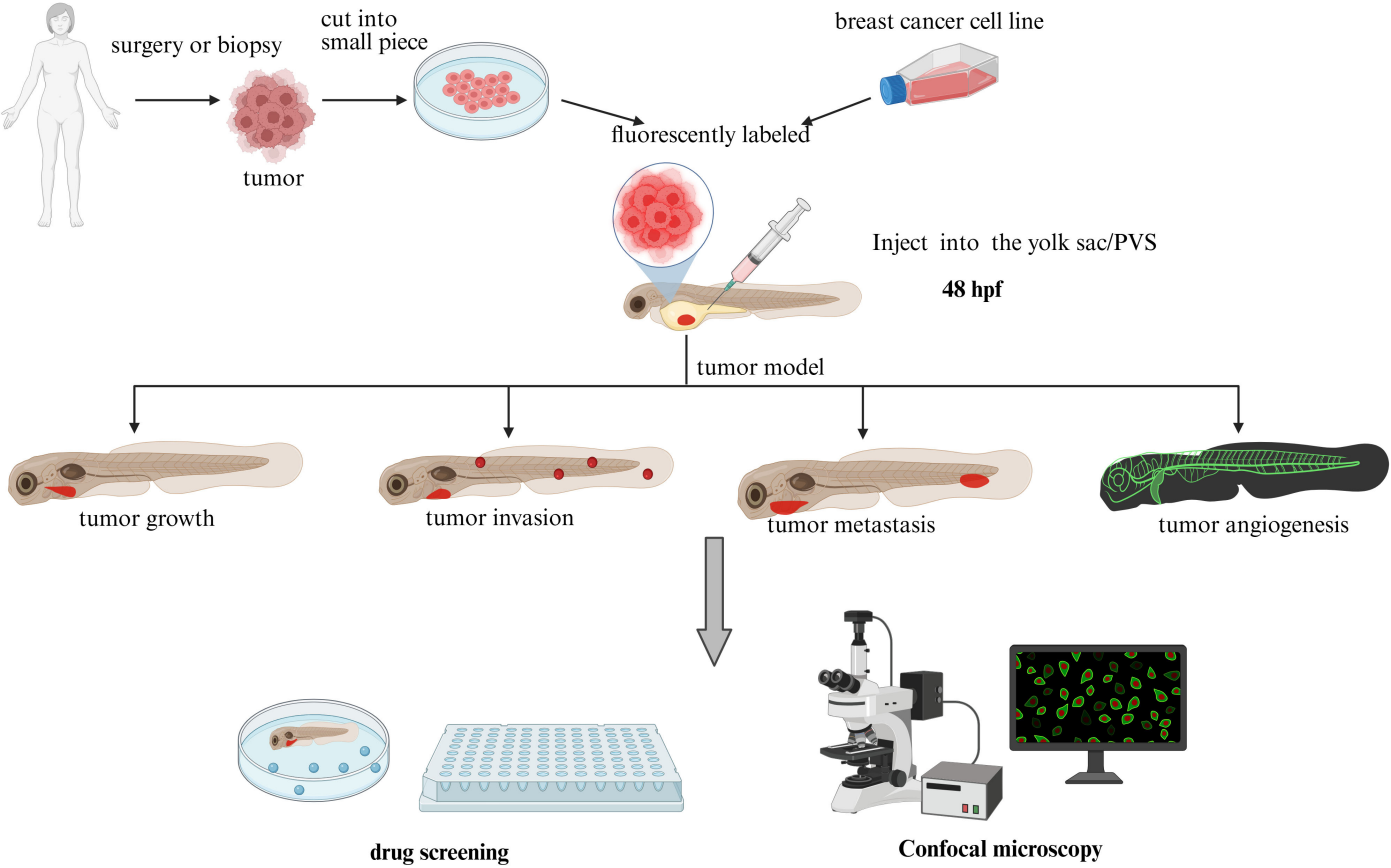
Zebrafish xenograft model (plot formation using Biorender.com).

Xenotransplantation serves as the primary methodology for developing zebrafish breast cancer models (**Figure 1**), involving engraftment of foreign tissues across species barriers. Embryonic xenotransplantation exploits the immunologically privileged window prior to 28 days post-fertilization, when adaptive immunity remains undeveloped (21), effectively preventing graft rejection. The miniature scale of zebrafish embryos permits tumor engraftment with as few as hundreds of cells, enabling single-cell resolution tracking of xenograft dynamics via intravital imaging. This low cellular inoculum closely recapitulates early tumorigenic events (22), mirroring initial stages of human cancer progression. This unique attribute makes zebrafish models particularly adept at modeling rare cell populations, including cancer stem cells (23–25) and circulating tumor cells (26, 27), with unprecedented spatiotemporal resolution. Cell dosage represents a critical experimental parameter requiring optimization in xenotransplantation protocols. Current protocols use cancer cell injections of 25-2,000 cells, with most research teams favoring injections of 50-200 (glioblastoma) (28) or 100-200 (colorectal cancer) (29) cells. We summarized the number of cells commonly used for different injection sites for most of the studies in this review as 200–300 for the yolk sac, 400 for the PVS, and 300–500 for the Duct of Cuvier. Vanda Póvoa et al. (30) used a zebrafish xenograft model to study the implantation efficiency of multiple human mammary glands. Breast cancer cells Hs578T showed high implantation rates (~95% implantation rate) whereas the implantation rate of MDA-MB-468 was less proliferative and less apoptotic. In actual studies, the number of cells implanted in successful xenografts varies by cell line, and optimization of each cell line is usually required (28).

Zebrafish xenograft models provide versatile implantation sites including: yolk sac (23–25), perivitelline space (PVS), common cardinal vein (CCV) (31, 32), and hindbrain ventricle (HBV). The yolk sac provides a lipid-rich microenvironment (cholesterol, phosphatidylcholine, triglycerides) (33) that enables high-contrast visualization of fluorescently labeled cells through standardized protocols. The yolk sac is the site of choice for the study of survival, cell division, proliferation and migration (34). This lipid matrix not only sustains xenograft viability but also potentiates tumor proliferation through nutrient enrichment. The perivitelline space (PVS), located between the periderm and the yolk syncytial layer, serves as a key site for xenografting cancer cells in zebrafish embryos. This model enables real-time study of neoangiogenesis dynamics and metastatic cascades (35, 36), as well as quantitative assessment of primary tumor regression in response to therapies (37, 38). Despite its experimental advantages, PVS microinjection requires advanced technical proficiency compared to yolk sac procedures (39). Common cardinal vein (CCV) or duct of Cuvier injection enables direct intravascular delivery (40) of tumor cells, facilitating real-time monitoring of metastatic processes (41). This technique demands exceptional microsurgical expertise, particularly in adult zebrafish models with reduced vascular lumen diameters (42). The hindbrain ventricle (HBV), characterized by dense vascularization, provides an optimal platform for investigating hematogenous metastasis mechanisms (43). However, the procedure demands submicron-level spatial precision during microinjection, as technical inaccuracies may induce structural damage to adjacent neural architectures. Optimal site selection requires careful consideration of microenvironmental relevance versus technical feasibility, depending on specific research objectives (e.g., metastatic mechanisms vs. high-throughput drug screening).

Xenografts originate from two primary sources: established cancer cell lines or patient-derived specimens (PDX, Patient-Derived Xenografts) obtained through surgical resection or biopsy. Standard preclinical workflows predominantly employ commercially available immortalized cell lines for tumor model establishment. The zebrafish xenograft literature documents multiple BC cell line options, necessitating careful selection based on specific research objectives. TNBC studies demonstrate preferential use of MDA-MB-231 (58% prevalence) (24, 25), followed by MDA-MB-468 (22%) (44), with BT549, CAL-148, HCC38, HCC70, MDA-MB-436, and MDA-MB-453 collectively accounting for <20% utilization. However, chronic 2D culture on plastic substrates coupled with repeated freeze-thaw cycles drives genetic drift and phenotypic convergence, compromising clinical translatability of findings (41). These limitations underscore the necessity of PDX models that preserve original tumor heterogeneity and drug response profiles. Contemporary precision oncology prioritizes individualized therapeutic strategies over conventional subtype classification, driven by recognition of intra-/inter-tumoral heterogeneity. Emerging PDX-zebrafish platforms now enable *in vivo* assessment of tumor progression dynamics and drug sensitivity profiles, directly informing personalized treatment regimens. Innovative approaches incorporating three-dimensional (3D) spheroid models (45) further enhance physiological relevance. Ambrosio et al. (45) demonstrated that high-glucose (HG) conditions potentiate mammary adipose tissue-derived mesenchymal stromal/stem cell (MAT-MSC)-mediated BC cell invasion through zebrafish xenograft studies. Zebrafish xenografts of HG-primed MCF7/MAT-MSC spheroids exhibited significantly increased metastatic dissemination, confirming microenvironment-mediated pro-invasive crosstalk. These advances position zebrafish models as transformative tools in precision oncology research.

## 3 Application of zebrafish breast cancer model

The standard workflow for utilizing zebrafish models in breast cancer (BC) research involves dissociating tumor cells into single-cell suspensions, labeling them with CM-Dil (red fluorescence) (11), and microinjecting the labeled cells into target sites (e.g., yolk sac, perivitelline space) of anesthetized *Tg(fli1a:EGFP)* embryos (46). Tumor burden is quantified pre- and post-treatment via semi-automated fluorescence intensity segmentation (i.e., FIJI/ImageJ) of confocal microscopy images (47). Metastatic progression is assessed through 3D reconstruction of Z-stacked image series and quantification of the number of disseminated tumor cells to the caudal hematopoietic plexus (48–50). Key quantitative endpoints include: (1) Tumor volume (mm³), calculated from XYZ-axis measurements; (2) Metastatic index (scored 0–4); (3) Angiogenic sprouting density (vessels/mm²); (4) Kaplan-Meier survival analysis with median survival duration. The zebrafish model’s high-throughput compatibility and optical transparency establish it as a premier platform for *in vivo* investigation of metastatic cascades, neovascular dynamics, and preclinical drug efficacy.

### 3.1 Invasion and metastasis of tumor cells

Metastatic dissemination constitutes a pathobiological cascade involving discrete yet interconnected molecular and cellular events (51). Deciphering the molecular drivers of metastatic competence enables rational development of targeted therapies and optimization of therapeutic strategies. TNBC is uniquely clinically aggressive, manifesting itself in early and high frequency of distant metastases, with a particular predilection for metastases to the lungs, brain, and skeletal system (4, 52). To dissect these mechanisms, sophisticated metastasis models in zebrafish now permit high-resolution interrogation of stromal determinants and molecular mediators governing metastatic tropism, leveraging advanced live imaging and single-cell tracking technologies (53).

#### 3.1.1 Zebrafish disease model for breast cancer brain metastasis

Zebrafish transparent embryos facilitate real-time observation of tumor cell-blood-brain barrier (BBB) interactions, while their BBB structure exhibits high conservation with humans (54).Current treatment options for breast cancer with brain metastasis remain limited, as the molecular mechanisms underlying breast cancer cell infiltration into the brain are incompletely understood. The nuclear respiratory factor 1 (NRF1) transcription factor has been shown to exhibit high activity in various human tumors, with aberrant expression contributing to the acquisition of breast cancer stem cell (BCSC) properties (55). Inhibitor of differentiation 3 (ID3) is a transcriptional regulatory protein that induces pluripotent endothelial stem cells (ESCs). Zebrafish embryo xenograft experiments revealed that ID3-overexpressing ESCs not only support BCSC tumor spheroid growth but also direct these spheroids toward the zebrafish neural crest. These findings reveal a novel role for the ID3 and NRF1 (56), wherein ID3-expressing ESCs facilitate the homing of NRF1-expressing BCSCs to metastatic niches, likely promoting colonization, survival, and proliferation. This mechanistic insight provides critical foundations for preclinical evaluation of NRF1/ID3-targeting agents to prevent cerebral metastasis. Notably, TNBC frequently progresses to leptomeningeal metastasis, a condition with limited therapeutic options and poor prognosis, partly due to the absence of representative animal models. Gopal et al. (57) established the first triple-negative leptomeningeal disease (TNLMD) zebrafish model using translucent *casper (roy-/-; nacre-/-)* embryos, in which cells were microinjected into the fourth ventricle, followed by microscopic and histochemical analyses to confirm intracranial localization. Quantification of fluorescent TNBC xenografts via corrected total cell fluorescence (CTCF) analysis enabled time-resolved monitoring of tumor proliferation and secondary migration, with doxorubicin treatment suppressing proliferation and inducing apoptosis. These results validate the zebrafish TNLMD model as a powerful platform for multimodal analysis of TNBC progression and drug responses in leptomeningeal disease. Emerging therapeutic strategies include targeting the CD2/CD27 axis to inhibit M2 macrophage polarization in metastatic microenvironments (58), and disrupting the MUC5AC/cMET/CD44v6 metastasis cascade. Single-cell sequencing has further identified MUC5AC overexpression in HER2+ brain metastatic lesions, correlating with adverse patient outcomes (59). The integration of zebrafish models into drug discovery pipelines for breast cancer brain metastasis promises to accelerate therapeutic development and improve clinical management.

#### 3.1.2 Zebrafish xenograft model for breast cancer with bone metastasis

Approximately 70% of patients with metastatic breast cancer develop bone metastases (60–63), and their 5-year survival rate is significantly reduced to 10-29% (64, 65), with treatment resistance (66) being the main cause of poor prognosis. PDX models represent an emerging strategy to recapitulate clinical therapeutic responses. Mercatali et al. (41) cultured primary bone metastasis cultures from a 67-year-old patient, subsequently microinjecting these cells into 2-dpf *Tg(kdrl:mCherry)* zebrafish via the duct of Cuvier to assess metastatic behavior. CFSE green fluorescent labeling provided superior phenotypic resolution compared to conventional CM-DiI red staining in this model system. The PDX cells demonstrated vascular extravasation competence, ultimately colonizing the caudal hematopoietic tissue (CHT). These findings validate PDX-zebrafish models as transformative tools for clinical-translational metastasis research. SCUBE2 expression and secretion are associated with osteoblast differentiation and bone metastasis in human tumors, and both targeting Hedgehog signaling with Sonidegib and targeting SCUBE2 with neutralizing antibodies can effectively inhibit bone metastasis in multiple metastasis models (67). The zebrafish transplantation model can mimic the colonization process of breast cancer cells in bone tissue, and it is feasible to screen anti-bone metastasis drugs (e.g. neutralizing antibody targeting SCUBE2). While zebrafish xenograft models provide valuable insights into tumor cell migration to hematopoietic niches, it is essential to clarify their limitations in modeling human bone metastasis. Embryonic/larval zebrafish (≤7 dpf) lack mature mineralized bone structures (68). Bone mineralization density—a critical determinant of breast cancer osteotropism (69)—remains negligible in these stages, as quantified by micro-CT and Alizarin red staining (68). Consequently, models relying on the *caudal hematopoietic plexus* (CHP) primarily reflect hematopoietic niche colonization rather than true bone metastasis.

In recent years, a new generation of bone metastasis models based on 3D constructs (including tumor spheroids, organoids, and bioscaffolds) (70) has attracted much attention. These models effectively overcome the inherent shortcomings of traditional two-dimensional culture systems (lack of three-dimensional cellular microenvironment) and animal models (high cost, ethical controversy, and physiological differences between species), and provide a more physiologically relevant experimental platform for the study of breast cancer bone metastasis. The research team successfully constructed a 3D nanoclay-based bone metastasis *in vitro* model by integrating human-derived MSCs with breast cancer cell lines (e.g., MCF-7, MDA-MB-231) or patient-derived cell lines (NT013, NT023) (71). This model system can assess the metastatic potential of individualized breast cancer subtypes, and provide an efficient technical means for personalized drug screening in advanced breast cancer. Ravi et al. (72) also utilized this nanoclay scaffold platform to reveal, for the first time, the inhibitory effect of Rhodiola on breast cancer bone metastasis. The experimental data showed that its active components selectively induced apoptosis in bone-metastatic breast cancer cells while maintaining normal bone tissue activity, indicating its potential value as a targeted therapeutic agent for bone metastasis. It is worth noting that the integration and application of the zebrafish model (with the advantage of *in vivo* dynamic monitoring) and the 3D model (with the ability to reconstruct the pathological microenvironment) is expected to realize a breakthrough of technological complementarity in the field of analyzing the mechanism of breast cancer bone metastasis and evaluating the efficacy of drugs.

#### 3.1.3 Analysis of circulating tumor cells in patients with metastatic breast cancer

Circulating tumor cells (CTCs) and CTC clusters play pivotal roles in metastatic cascade through their detachment from primary tumors, survival in circulation, and targeted colonization. *In vitro* CTC models and *in vivo* metastasis models synergistically facilitate mechanistic investigations into metastatic cell behavior and secondary tumor formation. Martinez-Pena et al. (73) demonstrated that CTC clusters exhibit superior survival capacity and proliferative potential compared to single CTCs via integrated breast cancer models combining single CTC/CTC cluster simulations and zebrafish embryo xenografts, with molecular mechanisms linked to cell cycle pathway activation and stemness-associated gene upregulation. However, current cell models fail to fully recapitulate patient-derived CTC heterogeneity. Additionally, efficient capture and isolation of CTCs/clusters from cancer patient blood remains technically challenging. To overcome the technical challenges in isolating rare CTC populations, Reinhardt’s team (26) developed DanioCTC, an integrated platform combining diagnostic leukapheresis, Parsortix™ microfluidics, FACS sorting, and CellCelector™ micromanipulation for high-purity CTC enrichment from metastatic breast cancer patients. In the DanioCTC pipeline, purified CTCs are xenotransplanted into the Cuvier ducts of 2-dpf *Tg(osx:mCherry)* zebrafish embryos, enabling real-time tracking of metastatic dissemination. DanioCTC represents a breakthrough in studying individualized and rare CTC subpopulations during metastatic dissemination, significantly advancing our understanding of metastatic breast cancer biology and enabling targeted therapeutic development. Notably, CTC clusters are emerging as critical mediators of metastasis. Elucidating their molecular regulatory networks and microenvironmental interactions may unlock novel therapeutic strategies against metastatic progression.

#### 3.1.4 TGF-β family signaling in breast cancer progression

TGF-β exhibits a paradoxical role in breast cancer pathogenesis: During early tumorigenesis, it exerts growth-suppressive effects on normal and premalignant mammary epithelium, whereas advanced malignancies develop resistance to these tumor-suppressive signals, enabling TGF-β to drive oncogenic progression (74). This functional switch enables TGF-β to promote metastasis through both cell-autonomous mechanisms inducing epithelial-mesenchymal transition/EMT) and microenvironmental reprogramming [mediating immune evasion (75) and neoangiogenesis (76)]. Li et al. (77) demonstrated through zebrafish xenotransplantation that TGF-β signaling orchestrates breast cancer cell intravasation/extravasation dynamics and angiogenic niche formation. In their experimental paradigm, mCherry-labeled MDA-MB-231 cells were microinjected into the PVS or Cuvier ducts of *Casper* (roy^-/-^;nacre^-/-^) embryos with ubiquitous enhanced green fluorescent protein (EGFP) expression in vasculature, allowing real-time tracking of metastatic cell behavior and tumor-induced vasculogenesis via confocal time-lapse imaging. This methodology establishes zebrafish xenotransplantation as a robust platform for interrogating TGF-β pathway interventions on metastatic dissemination and tumor vascularization.

#### 3.1.5 CXCR4-CXCL12 axis in early TNBC metastasis

The CXCR4-CXCL12 chemokine axis directs cell migration during physiological and pathological processes, including breast cancer metastasis. Despite ongoing clinical trials targeting this axis, no TNBC-specific CXCR4 inhibitors have achieved clinical translation, highlighting the need for improved preclinical models. Tulotta et al. (78) employed zebrafish TNBC xenografts to reveal evolutionarily conserved ligand-receptor cross-reactivity: Human CXCR4^+^ tumor cells primed metastatic niche formation through engagement of zebrafish CXCL12 homologs at distal sites. This interspecies compatibility enabled pharmacological validation using IT1t - a small-molecule CXCR4 antagonist, which significantly reduced metastatic burden *in vivo* via disrupting CXCL12-guided chemotaxis. Their work not only validates zebrafish models for CXCR4 inhibitor screening but also provides mechanistic proof-of-concept for axis-targeted therapy in TNBC.

### 3.2 Tumor angiogenesis

The zebrafish xenotransplantation model capitalizes on its physiologically simplified circulatory system, optical transparency during embryogenesis, and transgenic fluorescent vascular reporters to enable real-time visualization of tumor-associated angiogenesis. Standardized protocols involve microinjecting tumor cells into 2-day post-fertilization (dpf) embryos, followed by longitudinal quantification of angiogenic activity (35). *In vivo* confocal imaging enables dynamic tracking of angiogenic mechanisms, including sprouting angiogenesis and directional endothelial migration (79). Clinically approved anti-angiogenic agents predominantly target the VEGF pathway, which is a key regulator of pathological angiogenesis due to its mitogenic and chemotactic effects on endothelial cells (80–82). Nevertheless, the spatiotemporal coordination between xenograft-derived pro-angiogenic signals and host immune cell interactions remains incompletely mapped. Zebrafish macrophages have previously been shown to be required for inflammatory lymphangiogenesis and expression of pro-angiogenic VEGF ligands (83). Britto et al. (84) employed a nitroreductase-mediated approach to ablate macrophages and neutrophils, with subsequent quantitative assessment of graft vascularization using angiogenesis scoring metrics. The ablation of macrophages, but not neutrophils, caused a strong reduction in tumor xenograft vascularization and time-lapse imaging demonstrated that tumor xenograft macrophages directly associated with the migrating tip of developing tumor blood vessels. Macrophages were also found to be required for angiogenesis in xenografts secreting VEGFA or overexpressing zebrafish VEGFAA, suggesting that zebrafish macrophages enhance VEGFA-driven tumor angiogenesis. The importance of macrophages for angiogenesis suggests that this model could be used to further investigate the interaction between myeloid cells and tumor angiogenesis. To investigate pro-angiogenic niche effects, Ghajar et al. (85) utilized mtp-null mutants—exhibiting ectopic subintestinal plexus hypervascularization at 3.5 dpf—as microenvironmental amplifiers for tumor engraftment studies. mCherry-labeled MDA-MB-231 cells were microinjected into the subintestinal space (SIS) of WT versus *mtp^-/-^* mutants. *mtp^-/-^* mutants displayed 4-fold higher neovascular bud density post-injection compared to WT controls. While WT-engrafted tumors showed limited expansion, *mtp^-/-^* mutants supported aggressive perivascular tumor growth, particularly at neovascular branch points. Collectively, these data validate the zebrafish xenograft platform as an exceptional model for dissecting tumor angiogenesis dynamics.

### 3.3 Cancer drug screening

While conventional cell-based assays remain the mainstay for initial anti-tumor drug evaluation, their predictive validity is constrained by the absence of physiological context (e.g., vascular networks, immune components), resulting in high attrition rates during clinical translation. In contrast, vertebrate models provide holistic assessment of therapeutic efficacy, organ-specific toxicity profiles, and pharmacokinetic/pharmacodynamic (PK/PD) parameters, yet murine (12) and primate models prove economically prohibitive for large-scale drug screening. The zebrafish PDX platform uniquely combines high-throughput capability (enabled by rapid embryogenesis, translucent body wall, and optical clarity) with mammalian-relevant pathophysiology, delivering clinically actionable drug sensitivity data within 7 days (8, 86) - 5-fold faster than murine xenografts. This model has successfully validated compounds targeting angiogenesis, invasion, and metastasis in BC (examples provided in **Table 1**), with emerging agents like atypical cannabinoids (87) showing potent activity against taxane-resistant phenotypes and providing new mechanistic insights into chemoresistance reversal.

**Table 1:** Therapeutic molecules screened using the zebrafish xenograft model.

Drug name	Target of action	Effects	BC cell types	Injection site	Number of cells	Incubation temperature	Zebrafish line	References
Curcumin	BCSC	antiproliferation	BT474	yolk sac	500	28°C/34°C	WT	(23)
Gomisin M2	BCSC	antiproliferation	MDA-MB-231 HCC1806	yolk sac	200-300	34°C	WT	(24)
PL-NP	BCSC	antiproliferation	MDA-MB-231	PVS	400	32°C	Tg (kdrl:EGFP)	(25)
atypical cannabinoid	/	antiproliferation	MDA-MB-231	yolk sac	75-150	34°C	WT	(87)
CAR-NK	/	antiproliferation	MDA-MB-231 MDA-MB-453	Duct of Cuvier	100	33°C	Tg (flk1:GFP)	(48)
Edelfosenano-emulsion	/	antiproliferation	MDA-MB-231	yolk sac	40	34°C	WT	(120)
proteasome inhibitor (PI)	/	antiproliferation	HCC1806 MCF-7	yolk sac	500-1000	34°C	/	(121)
IT1t	CXCR4	anti early metastasis	MDA-MB-231 MDA-MB-231-B	Duct of Cuvier	300-500	34°C	Tg(kdrl:EGFP) Tg(mpeg1:mCherry) Tg(mpeg1:EGFP)	(78)
TKRI inhibitor	TGF-β	anti-invasive metastasis	MCF-10A MDA-MB-231	Duct of Cuvier	400	33°C	Tg(fli1:GFP)	(88)
MMP inhibitor	TGF-β	anti-invasive metastasis	MCF-10A MDA-MB-231	Duct of Cuvier	400	33°C	Tg(fli1:GFP)	(88)
av antagonist	TGF-β	anti-invasive metastasis	MDA-MB-231	Duct of Cuvier	400	33°C	Tg(fli1:GFP)	(31)
UCHL1 inhibitor 6RK73	TGF-β	antimetastasis	MDA-MB-231	Duct of Cuvier	400	33°C	Tg(fli1:EGFP)	(32)
Matcha	/	antimetastasis	MDA-MB-468 MDA-MB-231	yolk sac	/	/	WT	(44)
PRMT5	GR	antimetastasis	MDA-MB-231	yolk sac	300	34°C	/	(122)
Fangjihuangqi	EMT	antimetastasis	MDA-MB-231	yolk sac	200	35°C	WT	(123)
Bevacizumab	VEGF	antiangiogenesis	/	yolk sac	/	28.5°C	Tg(fli1a:EGFP)	(97)
PI3K nanoparticle	PI3K	antiangiogenesis	MDA-MB-231	yolk sac	500	/	/	(98)
SKLB646	VEGFR2	antiangiogenesis	/	/	/	/	Tg(flk1:EGFP)	(99)
Emodin	VEGFA	antiangiogenesis	MDA-MB-231	/	/	28.5°C	Tg(fli-1a:EGFP)	(100)
Musketone	VEGF/PI3K/Akt/MAPK	antiangiogenesis	MDA-MB-231	PVS	3x10^4^	28.5°C	Tg(fli-1a:EGFP)	(101)
FAK	VEGFR2	antiangiogenesis	MDA-MB-468 MDA-MB-231	PVS	/	32.5°C	Tg(fli1:EGFP)	(124)

#### 3.3.1 Anti-TGF-β signaling pathway drugs

Zebrafish xenograft models have emerged as a cost-efficient platform for screening anti-TGF-β therapeutics against metastatic breast cancer. Drabsch et al. (88) established an innovative metastasis assay by microinjecting human breast cancer cells into duct of Cuvier, enabling real-time tracking of tumor cell invasion into the avascular caudal fin. Pharmacological interrogation using TGF-βRI kinase inhibitors [SB-431542 (89, 90), LY-294002 (91)], MMP inhibitor GM6001 (92), and Smad4 genetic ablation demonstrated that blockade of different parts of the TGF-β signaling pathway results in a significant reduction in breast cancer cell invasion and metastasis. The model’s versatility is further evidenced by successful evaluation of αv integrin antagonists (31), CXCR4 inhibitor IT1t (78), and UCHL1 inhibitor 6RK73 (32), collectively validating zebrafish as a premier system for TGF-β-targeted drug discovery.

#### 3.3.2 Anti-VEGF signaling pathway drugs

The zebrafish PVS (93, 94) implantation model enables quantitative assessment of tumor-induced angiogenesis through longitudinal monitoring of subintestinal vessel (SIV) sprouting at 48-hour post-implantation. This process is governed by the angiogenic switch mechanism, where dynamic equilibrium between pro-angiogenic (e.g., VEGF/VEGFR axis) and anti-angiogenic factors (e.g., p53-mediated pathways) determines neovascularization outcomes (95). While bevacizumab - a humanized anti-VEGF monoclonal antibody approved since 2004, its clinical efficacy is limited by compensatory mechanisms and acquired resistance (96). Zhang et al. (97) pioneered zebrafish-based anti-angiogenic drug evaluation, demonstrating bevacizumab inhibited the formation of subintestinal veins in zebrafish and mimicked the process of tumor angiogenesis *in vivo*. Zebrafish platform has facilitated development of therapeutics including: (1) PI3K inhibitor-loaded nanoparticles showing reduction in MDA-MB-231-induced angiogenesis (98); (2) SKLB646 (SRC/Raf/VEGFR2 multi-kinase inhibitor) suppressing intersegmental vessel formation (99). Emerging strategies focus on natural compounds like emodin (100) and muscone (musk ketone) (101), with the latter significantly attenuating VEGF-mediated signaling in zebrafish SIV. The optical transparency of zebrafish embryos coupled with transgenic vascular reporters (e.g., *fli1a:EGFP*) provides unparalleled spatiotemporal resolution for anti-angiogenic drug discovery.

#### 3.3.3 Screening of the anti-cancer drugs targeting breast cancer stem cells

Cancer stem cells (CSCs) possess three defining hallmarks: (1) self-renewal capacity, (2) multilineage differentiation potential, and (3) tumor-initiating ability, making them prime therapeutic targets for eradicating malignant clones. Therefore, targeting CSCs could be an important anti-cancer therapeutic strategy. Eguiara et al. (23) demonstrated CSC pathotropism using 2-dpf zebrafish xenografts, revealing that CSCs exhibited higher caudal engraftment efficiency compared to parental cell populations. Curcumin supplementation significantly impaired CSC migratory capacity and proliferation, establishing zebrafish as a dual-purpose platform for fundamental CSC biology and drug discovery. Yang et al. (24) further validated this approach through microinjection of 200–300 BCSC-enriched MDA-MB-231-GFP cells, showing that gomisin M2 significantly inhibited BCSC proliferation and mammosphere formation. Metabolic reprogramming studies identified elevated glycolytic flux in BCSCs as critical for maintaining stemness and chemoresistance, with Singh et al. (25) demonstrating that PL-NPs (piperlongumine nanoparticles) reversed this phenotype through inhibition of glutathione transferase GSTP 1 expression and upregulation of FBP 1 (a key metabolic enzyme for gluconeogenesis) expression in *Tg(kdrl:EGFP)* zebrafish. The PLGA-encapsulated formulation enhanced drug bioavailability while leveraging the Enhanced permeability and retention (EPR) effect for tumor-selective accumulation, addressing key pharmacokinetic limitations of free piperlongumine. These multidisciplinary approaches establish zebrafish models as indispensable tools for developing CSC-targeted therapies against TNBC.

#### 3.3.4 Humanized zebrafish models for evaluating CAR-NK immunotherapy and immune checkpoint blockade

The zebrafish model’s high fecundity and compatibility with microscale tumor inocula establish it as a premier platform for personalized therapeutic validation. Shankar et al. (48) established a dual-cell xenotransplantation paradigm in zebrafish, sequentially engrafting metastatic breast cancer cells and chimeric antigen receptor-natural killer (CAR-NK) cells into the duct of Cuvier to evaluate antitumor efficacy. Cancer cells preferentially colonized distal niches including the caudal hematopoietic tissue (CHT), mimicking organotropic metastasis to hematopoietic microenvironments. CAR-NK cells exhibited targeted tropism, infiltrating CHT microenvironments within 2.5 h post-injection (hpi) and rapidly eliminated individual cancer cells throughout the organism. This methodology enables rapid preclinical evaluation of CAR-NK cell therapies, demonstrating strong translational potential for personalized immunotherapy optimization. Beyond evaluating CAR-NK cell efficacy, zebrafish models offer unique advantages as humanized preclinical platforms for immunotherapy research. Through co-engraftment of patient-derived tumor cells and human immune components (e.g., NK cells) (102) into optically transparent casper mutant embryos, these models recapitulate critical aspects of the human tumor-immune microenvironment (TIME). This enables: (i) Real-time tracking of immune cell infiltration into primary tumors via confocal imaging of fluorescently labeled T cells/NK cells; (ii) Quantitative assessment of immune checkpoint inhibitor (ICI) responses (e.g., anti-PD-1 induced T-cell reactivation); (iii) Combinatorial therapy screening – exemplified by synergistic CAR-NK/ICI (103, 104) regimens that remodel immunosuppressive microenvironments. The model’s capacity for longitudinal intravital imaging at single-cell resolution provides unprecedented insight into dynamic immune-tumor interactions (50) unattainable in murine systems.

## 4 Limitations of using the zebrafish xenotransplantation model

There are also some limitations to the use of the zebrafish model in breast cancer research. Although zebrafish is homologous to many mammalian pathways and organ systems, it does lack several mammalian tissues (lung, breast, and prostate) (105), but may be able to “add” desired cells or growth signals to allow normal growth cues associated with *in situ* injections (34). For example, transgenic fish (15) expressing human growth factors, receptors and/or cytokines could be generated to enhance implantation and tumor growth.

There are inherent temperature differences between zebrafish and human systems that may influence tumor cell proliferation rates and zebrafish physiology. Zebrafish embryos develop optimally at 27°C-28°C (39, 106), whereas human tumor cells thrive at 37°C (107), a temperature required for mammalian cell viability. To address this discrepancy, xenograft experiments methodologically implement a compromise temperature that balances optimal growth conditions in the host with the metabolic requirements of the tumor cells. This temperature must be sufficiently high to sustain proliferation of xenografted cells while maintaining embryo viability and preventing developmental defects. In recent years, the standard temperature for performing xenograft assays in the literature has been increased to 34°C at the expense of shortening the incubation time to 3 to 6 days post-injection (dpi) (39). Incubation temperatures and incubation times remain unbalanced. In an experimental design comparing incubation temperatures (28, 34 and 36°C), Pablo et al. (108) systematically evaluated morphological abnormalities and developmental effects in injected and control embryos at multiple time points. Their results showed that incubation at 36°C for 48 h was most favorable for xenograft survival, and no significant morphological or metabolic disturbances were observed in either host embryos or tumor cells, allowing them to proliferate near their respective optimal temperatures. Protocols are adapted to tumor cell requirements: while 34°C (39) remains widely used for viability balance, temperature escalation (35-36°C) (109, 110) may be implemented for short-term drug efficacy assays with mortality controls.

Zebrafish xenograft models faithfully replicate conserved metastatic mechanisms documented in murine models, including evolutionarily maintained signaling pathways (e.g., HTR2A/2B-mediated EMT induction (111)) and prototypical metastatic cascades such as hematogenous dissemination. However, phylogenetic divergences in immunoinhibitory checkpoint regulation and organotropic metastasis predilection compel the implementation of complementary methodology combining both model organisms to optimize experimental validity and translational relevance (12, 51). Paul et al. (112) demonstrated that cell line pairs that preferentially target bone marrow and brain niches in mice show similar targeting in the larval fish by 5 dpi following injection to the circulatory system. The authors (112) validated the conservation of organotropism across species by injecting zebrafish with mouse-derived 4T1Br4 (brain-homing) and 4T1BM2 (bone marrow-tropic) cells, and observed metastatic targeting patterns that aligned with murine experimental data. The optical transparency of zebrafish embryos enables real-time visualization of tumor cell-microenvironment interactions, a technical challenge in opaque mammalian models. Fluorescent lineage tracing in zebrafish has enabled precise tracking of metastatic processes, including vascular migration and micrometastatic colonization (51). Whereas zebrafish larval models enable rapid metastasis assays (days-scale), murine models require extended observation periods (weeks to months) (113). Crucially, murine models maintain indispensable translational relevance by reconstituting humanized adaptive immune microenvironments and supporting evolutionarily conserved myeloid differentiation pathways that generate metastasis-associated macrophages (MAMs) (114), a process incompletely modeled in zebrafish. While zebrafish excel in modeling early metastatic dissemination, murine models remain superior for studying organ-specific metastases [e.g., bone metastasis (115)] and immune microenvironment dynamics, as evidenced by studies of breast cancer bone metastasis employing transgenic mouse models [e.g., MMTV-PyMT (116)]. Recent methodological advances now enable implantation of patient-derived tumor samples in zebrafish, significantly improving human TME complexity recapitulation and demonstrating high treatment response predictability (117). As demonstrated by the results of Mendes et al. (118), the zebrafish TNBC xenograft model effectively differentiates between sensitivity to anthracyclines and paclitaxel, and the efficacy of combination chemotherapy is superior to that of single-agent treatment. The BC zAvatars model can be efficiently established (including early and late stage tumors), retains key biomarkers (e.g., ER), and its therapeutic response is highly concordant with the patients’ clinical outcomes (18/18 perfect match). The model also accurately reflects tumor staging and micrometastatic characteristics, providing a basis for personalized decisions on adjuvant treatment intensity and monitoring regimens. Nevertheless, key limitations persist (51).

Furthermore, while implanting a small number of transplanted cells (around 100–200 per embryo) can ensure successful engraftment, it may not necessarily include cancer-driving stem cells, which are indicative of the genetic diversity present in human tumors. Another disadvantage of embryo xenotransplantation is that the developing embryo provides a different biological microenvironment than the adult human body, and therefore the transplanted cancer cells are surrounded by new signals, which may have implications for cancer biology. The delayed maturation of zebrafish adaptive immunity imposes temporal limitations on longitudinal studies of tumor immunobiology or therapeutic response kinetics. While the immunodeficient larval stage facilitates xenotransplantation, it precludes investigation of adaptive immunity’s dual roles in tumor suppression and therapy potentiation (34). Although zebrafish are useful for screening TME-centered chemicals, mammalian models remain necessary to develop these findings into drugs with favorable pharmacokinetic/pharmacodynamic properties (119). From a technical point of view, most studies have been developed by using membrane and cytoplasmic dyes to color tumor cells. These dyes do not correctly distinguish between dead and living cells, leading to biased overestimation of tumor mass and growth. As the zebrafish model becomes more widely used, the limitations will be dissolved.

## 5 Discussion and perspectives

Zebrafish have emerged as a valuable *in vivo* model for human disease research, particularly in oncology, due to their unique biological advantages. Zebrafish xenotransplantation represents a powerful experimental strategy that enables cost-efficient, high-throughput investigation of human malignancies, overcoming the limitations of small sample sizes encountered in mammalian models. Nevertheless, critical knowledge gaps persist, including the development of immunocompromised zebrafish strains resistant to tumor rejection and the long-term viability of xenografts. These gaps underscore the urgent need for further research, especially given the clinical challenges faced by patients with TNBC. Patients with TNBC—who derive limited benefit from anti-HER2 or endocrine therapies, exhibit poor five-year survival rates and elevated early recurrence risk. Elucidating the molecular mechanisms underlying breast cancer progression through systematic investigation is essential for identifying novel therapeutic targets to improve clinical outcomes in this high-risk patient population. Zebrafish have emerged as an indispensable tool in this endeavor, with accumulating evidence reviewed herein supporting the transformative potential of zebrafish xenografts in both fundamental breast cancer research and therapeutic development. The advent of 3D multicellular co-culture platforms enables precise dissection of tumor-stroma crosstalk. Integrating these systems with zebrafish xenografts could map spatial-temporal interactions between malignant cells and specific TME components. Future efforts should primarily concentrate on standardizing zebrafish model protocols and validating their clinical translatability. This can be achieved through strategies like integrating organoid co-culture systems, ultimately aiming to enhance predictive accuracy and ensure a smoother transition from bench to bedside. Consequently, leveraging zebrafish models in high-throughput drug screening pipelines may accelerate the translation of preclinical discoveries into targeted therapies for breast cancer patients. Taken together, these advancements position zebrafish as a cornerstone model in precision oncology and personalized medicine paradigms.

## References

[1] Bray F Laversanne M Sung H Ferlay J Siegel RL Soerjomataram I. Global cancer statistics 2022: GLOBOCAN estimates of incidence and mortality worldwide for 36 cancers in 185 countries. CA Cancer J Clin. (2024) 74:229–63. doi: 10.3322/caac.21834, PMID: 38572751

[2] Siegel RL Giaquinto AN Jemal A. Cancer statistics, 2024. CA Cancer J Clin. (2024) 74:12–49. doi: 10.3322/caac.21820, PMID: 38230766

[3] Al-Mahmood S Sapiezynski J Garbuzenko OB Minko T. Metastatic and triple-negative breast cancer: challenges and treatment options. Drug Delivery Transl Res. (2018) 8:1483–507. doi: 10.1007/s13346-018-0551-3, PMID: 29978332 PMC6133085

[4] Grote I Poppe A Lehmann U Christgen M Kreipe H Bartels S. Frequency of genetic alterations differs in advanced breast cancer between metastatic sites. Genes Chromosomes Cancer. (2024) 63:e23199. doi: 10.1002/gcc.23199, PMID: 37672607

[5] Deng X Hua K Munankarmy A Luo Q Wang X Fang L. E2F1-mediated ectopic expression of PP1A promotes breast cancer progression via activation of YAP1. Int J Biochem Cell Biol. (2023) 157:106389. doi: 10.1016/j.biocel.2023.106389, PMID: 36787863

[6] Marchio V Augimeri G Morelli C Vivacqua A Giordano C Catalano S. Omega-3 fatty acids: molecular weapons against chemoresistance in breast cancer. Cell Mol Biol Lett. (2025) 30:11. doi: 10.1186/s11658-025-00694-x, PMID: 39863855 PMC11762563

[7] Zhao Y Tan H Zhang J Zhan D Yang B Hong S. Developing liver-targeted naringenin nanoparticles for breast cancer endocrine therapy by promoting estrogen metabolism. J Nanobiotechnology. (2024) 22:122. doi: 10.1186/s12951-024-02356-0, PMID: 38504208 PMC10953142

[8] Hu C Sun L Chen J Lyu Z Yuan C Jiang X. Advantages of the zebrafish tumor xenograft model: the evaluation of efficacy in cancer therapy and the application to the study of lncRNAs. Front Immunol. (2024) 15:1483192. doi: 10.3389/fimmu.2024.1483192, PMID: 39403375 PMC11471560

[9] Roy S Saha S Dhar D Chakraborty P Singha Roy K Mukherjee C. Molecular crosstalk between CUEDC2 and ERα influences the clinical outcome by regulating mitosis in breast cancer. Cancer Gene Ther. (2022) 29:1697–706. doi: 10.1038/s41417-022-00494-x, PMID: 35732909

[10] Zhao S Huang J Ye J. A fresh look at zebrafish from the perspective of cancer research. J Exp Clin Cancer Res. (2015) 34:80. doi: 10.1186/s13046-015-0196-8, PMID: 26260237 PMC4531851

[11] Marques IJ Weiss FU Vlecken DH Nitsche C Bakkers J Lagendijk AK. Metastatic behaviour of primary human tumours in a zebrafish xenotransplantation model. BMC Cancer. (2009) 9:128. doi: 10.1186/1471-2407-9-128, PMID: 19400945 PMC2697170

[12] Singhal SS Garg R Mohanty A Garg P Ramisetty SK Mirzapoiazova T. Recent advancement in breast cancer research: insights from model organisms-mouse models to zebrafish. Cancers (Basel). (2023) 15:2961. doi: 10.3390/cancers15112961, PMID: 37296923 PMC10252042

[13] Zhu J Yang J Wen H Wang M Zheng X Zhao J. Expression and functional analysis of fam76b in zebrafish. Fish Shellfish Immunol. (2023) 142:109161. doi: 10.1016/j.fsi.2023.109161, PMID: 37838209

[14] Vierstraete J Willaert A Vermassen P Coucke PJ Vral A Claes KBM. Accurate quantification of homologous recombination in zebrafish: brca2 deficiency as a paradigm. Sci Rep. (2017) 7:16518. doi: 10.1038/s41598-017-16725-3, PMID: 29184099 PMC5705637

[15] Cayuela ML Claes KBM Ferreira MG Henriques CM van Eeden F Varga M. The zebrafish as an emerging model to study DNA damage in aging, cancer and other diseases. Front Cell Dev Biol. (2018) 6:178. doi: 10.3389/fcell.2018.00178, PMID: 30687705 PMC6335974

[16] Basten SG Davis EE Gillis AJ van Rooijen E Stoop H Babala N. Mutations in LRRC50 predispose zebrafish and humans to seminomas. PLoS Genet. (2013) 9:e1003384. doi: 10.1371/journal.pgen.1003384, PMID: 23599692 PMC3627517

[17] Lam SH Wu YL Vega VB Miller LD Spitsbergen J Tong Y. Conservation of gene expression signatures between zebrafish and human liver tumors and tumor progression. Nat Biotechnol. (2006) 24:73–5. doi: 10.1038/nbt1169, PMID: 16327811

[18] Mizgireuv IV Revskoy SY. Transplantable tumor lines generated in clonal zebrafish. Cancer Res. (2006) 66:3120–5. doi: 10.1158/0008-5472.CAN-05-3800, PMID: 16540662

[19] Spitsbergen JM Tsai HW Reddy A Miller T Arbogast D Hendricks JD. Neoplasia in zebrafish (Danio rerio) treated with N-methyl-N’-nitro-N-nitrosoguanidine by three exposure routes at different developmental stages. Toxicol Pathol. (2000) 28:716–25. doi: 10.1177/019262330002800512, PMID: 11026608

[20] Spitsbergen JM Tsai HW Reddy A Miller T Arbogast D Hendricks JD. Neoplasia in zebrafish (Danio rerio) treated with 7,12-dimethylbenz[a]anthracene by two exposure routes at different developmental stages. Toxicol Pathol. (2000) 28:705–15. doi: 10.1177/019262330002800511, PMID: 11026607

[21] Lam SH Chua HL Gong Z Lam TJ Sin YM. Development and maturation of the immune system in zebrafish, Danio rerio: a gene expression profiling, in *situ* hybridization and immunological study. Dev Comp Immunol. (2004) 28:9–28. doi: 10.1016/S0145-305X(03)00103-4, PMID: 12962979

[22] Lal S La Du J Tanguay RL Greenwood JA. Calpain 2 is required for the invasion of glioblastoma cells in the zebrafish brain microenvironment. J Neurosci Res. (2012) 90:769–81. doi: 10.1002/jnr.22794, PMID: 22183788 PMC3274595

[23] Eguiara A Holgado O Beloqui I Abalde L Sanchez Y Callol C. Xenografts in zebrafish embryos as a rapid functional assay for breast cancer stem-like cell identification. Cell Cycle. (2011) 10:3751–7. doi: 10.4161/cc.10.21.17921, PMID: 22033190

[24] Yang Y Hao E Pan X Tan D Du Z Xie J. Gomisin M2 from Baizuan suppresses breast cancer stem cell proliferation in a zebrafish xenograft model. Aging (Albany NY). (2019) 11:8347–61. doi: 10.18632/aging.102323, PMID: 31612865 PMC6814583

[25] Singh P Sen K Sa P Khuntia A Raghav SK Swain RK. Piperlongumine based nanomedicine impairs glycolytic metabolism in triple negative breast cancer stem cells through modulation of GAPDH & FBP1. Phytomedicine. (2024) 123:155181. doi: 10.1016/j.phymed.2023.155181, PMID: 38091824

[26] Reinhardt F Coen L Rivandi M Franken A Setyono ESA Lindenberg T. DanioCTC: analysis of circulating tumor cells from metastatic breast cancer patients in zebrafish xenografts. Cancers (Basel). (2023) 15:5411. doi: 10.3390/cancers15225411, PMID: 38001672 PMC10670801

[27] Hurtado P Martínez-Pena I Yepes-Rodríguez S Bascoy-Otero M Abuín C Fernández-Santiago C. Modelling metastasis in zebrafish unveils regulatory interactions of cancer-associated fibroblasts with circulating tumor cells. Front Cell Dev Biol. (2023) 11:1076432. doi: 10.3389/fcell.2023.1076432, PMID: 36949770 PMC10025339

[28] Pliakopanou A Antonopoulos I Darzenta N Serifi I Simos YV Katsenos AP. Glioblastoma research on zebrafish xenograft models: a systematic review. Clin Transl Oncol. (2024) 26:311–25. doi: 10.1007/s12094-023-03258-7, PMID: 37400666 PMC10810942

[29] Fontana CM Van Doan H. Zebrafish xenograft as a tool for the study of colorectal cancer: a review. Cell Death Dis. (2024) 15:23. doi: 10.1038/s41419-023-06291-0, PMID: 38195619 PMC10776567

[30] Póvoa V Rebelo de Almeida C Maia-Gil M Sobral D Domingues M Martinez-Lopez M. Innate immune evasion revealed in a colorectal zebrafish xenograft model. Nat Commun. (2021) 12:1156. doi: 10.1038/s41467-021-21421-y, PMID: 33608544 PMC7895829

[31] Li Y Drabsch Y Pujuguet P Ren J van Laar T Zhang L. Genetic depletion and pharmacological targeting of αv integrin in breast cancer cells impairs metastasis in zebrafish and mouse xenograft models. Breast Cancer Res. (2015) 17:28. doi: 10.1186/s13058-015-0537-8, PMID: 25849225 PMC4381510

[32] Liu S González-Prieto R Zhang M Geurink PP Kooij R Iyengar PV. Deubiquitinase activity profiling identifies UCHL1 as a candidate oncoprotein that promotes TGFβ-induced breast cancer metastasis. Clin Cancer Res. (2020) 26:1460–73. doi: 10.1158/1078-0432.CCR-19-1373, PMID: 31857432 PMC7611208

[33] Fraher D Sanigorski A Mellett NA Meikle PJ Sinclair AJ Gibert Y. Zebrafish embryonic lipidomic analysis reveals that the yolk cell is metabolically active in processing lipid. Cell Rep. (2016) 14:1317–29. doi: 10.1016/j.celrep.2016.01.016, PMID: 26854233

[34] Veinotte CJ Dellaire G Berman JN. Hooking the big one: the potential of zebrafish xenotransplantation to reform cancer drug screening in the genomic era. Dis Model Mech. (2014) 7:745–54. doi: 10.1242/dmm.015784, PMID: 24973744 PMC4073264

[35] Nicoli S Presta M. The zebrafish/tumor xenograft angiogenesis assay. Nat Protoc. (2007) 2:2918–23. doi: 10.1038/nprot.2007.412, PMID: 18007628

[36] Drabsch Y Snaar-Jagalska BE Ten Dijke P. Fish tales: The use of zebrafish xenograft human cancer cell models. Histol Histopathol. (2017) 32:673–86. doi: 10.14670/HH-11-853, PMID: 27933602

[37] Fieuws C Bek JW Parton B De Neef E De Wever O Hoorne M. Zebrafish avatars: toward functional precision medicine in low-grade serous ovarian cancer. Cancers (Basel). (2024) 16:1812. doi: 10.3390/cancers16101812, PMID: 38791891 PMC11120355

[38] van den Bosch QCC Kiliç E Brosens E. Uveal melanoma zebrafish xenograft models illustrate the mutation status-dependent effect of compound synergism or antagonism. Invest Ophthalmol Vis Sci. (2024) 65:26. doi: 10.1167/iovs.65.10.26, PMID: 39163035 PMC11346061

[39] Cabezas-Sáinz P Pensado-López A Sáinz B Jr. Sánchez L. Modeling cancer using zebrafish xenografts: drawbacks for mimicking the human microenvironment. Cells. (2020) 9:1978. doi: 10.3390/cells9091978, PMID: 32867288 PMC7564051

[40] Tulotta C He S Chen L Groenewoud A van der Ent W Meijer AH. Imaging of human cancer cell proliferation, invasion, and micrometastasis in a zebrafish xenogeneic engraftment model. Methods Mol Biol. (2016) 1451:155–69. doi: 10.1007/978-1-4939-3771-4_11, PMID: 27464807

[41] Mercatali L La Manna F Groenewoud A Casadei R Recine F Miserocchi G. Development of a patient-derived xenograft (PDX) of breast cancer bone metastasis in a zebrafish model. Int J Mol Sci. (2016) 17:1375. doi: 10.3390/ijms17081375, PMID: 27556456 PMC5000770

[42] He F Tu L Chan L Leung A Sun X. Optimized intravenous injection in adult zebrafish. J Vis Exp. (2024) 20:214. doi: 10.3791/67463, PMID: 39760378

[43] Lawrence JM Tan SH Kim DC Tan KE Schroeder SE Yeo KS. Diverse engraftment capability of neuroblastoma cell lines in zebrafish larvae. Zebrafish. (2024) 21:385–93. doi: 10.1089/zeb.2024.0160, PMID: 39316469 PMC11876807

[44] Sokary S Zakaria Z Bawadi H Al-Asmakh M. Testing the anticancer effect of matcha using zebrafish as an animal model. Nutrients. (2023) 15:2369. doi: 10.3390/nu15102369, PMID: 37242252 PMC10220593

[45] Ambrosio MR Mosca G Migliaccio T Liguoro D Nele G Schonauer F. Glucose enhances pro-tumorigenic functions of mammary adipose-derived mesenchymal stromal/stem cells on breast cancer cell lines. Cancers (Basel). (2022) 14:5421. doi: 10.3390/cancers14215421, PMID: 36358839 PMC9655059

[46] Nicoli S Ribatti D Cotelli F Presta M. Mammalian tumor xenografts induce neovascularization in zebrafish embryos. Cancer Res. (2007) 67:2927–31. doi: 10.1158/0008-5472.CAN-06-4268, PMID: 17409396

[47] Rebelo de Almeida C Mendes RV Pezzarossa A Gago J Carvalho C Alves A. Zebrafish xenografts as a fast screening platform for bevacizumab cancer therapy. Commun Biol. (2020) 3:299. doi: 10.1038/s42003-020-1015-0, PMID: 32523131 PMC7286887

[48] Murali Shankar N Ortiz-Montero P Kurzyukova A Rackwitz W Künzel SR Wels WS. Preclinical assessment of CAR-NK cell-mediated killing efficacy and pharmacokinetics in a rapid zebrafish xenograft model of metastatic breast cancer. Front Immunol. (2023) 14:1254821. doi: 10.3389/fimmu.2023.1254821, PMID: 37885894 PMC10599014

[49] Hemsing AL Førde JL Reikvam H Herfindal L. The Rac1-inhibitor EHop-016 attenuates AML cell migration and enhances the efficacy of daunorubicin in MOLM-13 transplanted zebrafish larvae. Transl Oncol. (2024) 40:101876. doi: 10.1016/j.tranon.2024.101876, PMID: 38185059 PMC10818244

[50] Arner A Ettinger A Blaser BW Schmid B Jeremias I Rostam N. *In vivo* monitoring of leukemia-niche interactions in a zebrafish xenograft model. PLoS One. (2024) 19:e0309415. doi: 10.1371/journal.pone.0309415, PMID: 39213296 PMC11364250

[51] Martínez-López MF López-Gil JF. Small fish, big answers: zebrafish and the molecular drivers of metastasis. Int J Mol Sci. (2025) 26:871. doi: 10.3390/ijms26030871, PMID: 39940643 PMC11817282

[52] Kan JY Lee HC Hou MF Tsai HP Jian SF Chang CY. Metabolic shifts in lipid utilization and reciprocal interactions within the lung metastatic niche of triple-negative breast cancer revealed by spatial multi-omics. Cell Death Dis. (2024) 15:899. doi: 10.1038/s41419-024-07205-4, PMID: 39695088 PMC11655832

[53] Chen X Li Y Yao T Jia R. Benefits of zebrafish xenograft models in cancer research. Front Cell Dev Biol. (2021) 9:616551. doi: 10.3389/fcell.2021.616551, PMID: 33644052 PMC7905065

[54] Li XG Niu C Lu P Wan HW Jin WD Wang CX. Screening and identification of hub-gene associated with brain metastasis in breast cancer. Med (Baltimore). (2023) 102:e32771. doi: 10.1097/MD.0000000000032771, PMID: 36800575 PMC9935999

[55] Das JK Felty Q Poppiti R Jackson RM Roy D. Nuclear respiratory factor 1 acting as an oncoprotein drives estrogen-induced breast carcinogenesis. Cells. (2018) 7:234. doi: 10.20944/preprints201809.0183.v1, PMID: 30486409 PMC6316306

[56] Das JK Deoraj A Roy D Felty Q. Brain infiltration of breast cancer stem cells is facilitated by paracrine signaling by inhibitor of differentiation 3 to nuclear respiratory factor 1. J Cancer Res Clin Oncol. (2022) 148:2881–91. doi: 10.1007/s00432-022-04026-w, PMID: 35678885 PMC11801135

[57] Gopal U Monroe JD Marudamuthu AS Begum S Walters BJ Stewart RA. Development of a triple-negative breast cancer leptomeningeal disease model in zebrafish. Cells. (2023) 12:995. doi: 10.3390/cells12070995, PMID: 37048068 PMC10093412

[58] Huang G Wu Y Gan H Chu L. Overexpression of CD2/CD27 could inhibit the activation of nitrogen metabolism pathways and suppress M2 polarization of macrophages, thereby preventing brain metastasis of breast cancer. Transl Oncol. (2023) 37:101768. doi: 10.1016/j.tranon.2023.101768, PMID: 37666207 PMC10480780

[59] Maurya SK Jaramillo-Gómez JA Rehman AU Gautam SK Fatima M Khan MA. Mucin 5AC Promotes Breast Cancer Brain Metastasis through cMET/CD44v6. Clin Cancer Res. (2025) 31:921–35. doi: 10.1158/1078-0432.CCR-24-1977, PMID: 39760691 PMC11882111

[60] Roodman GD. Mechanisms of bone metastasis. N Engl J Med. (2004) 350:1655–64. doi: 10.1056/NEJMra030831, PMID: 15084698

[61] Coleman RE. Metastatic bone disease: clinical features, pathophysiology and treatment strategies. Cancer Treat Rev. (2001) 27:165–76. doi: 10.1053/ctrv.2000.0210, PMID: 11417967

[62] Mundy GR. Metastasis to bone: causes, consequences and therapeutic opportunities. Nat Rev Cancer. (2002) 2:584–93. doi: 10.1038/nrc867, PMID: 12154351

[63] Ibrahim T Mercatali L Amadori D. A new emergency in oncology: Bone metastases in breast cancer patients (Review). Oncol Lett. (2013) 6:306–10. doi: 10.3892/ol.2013.1372, PMID: 24137321 PMC3789111

[64] Hu H Hu X Liang Z Yang W Li S Li D. Diagnostic performance of (18)F−FDG PET/CT vs. (18)F−NaF PET/CT in breast cancer with bone metastases: An indirect comparative meta−analysis. Oncol Lett. (2024) 28:546. doi: 10.3892/ol.2024.14679, PMID: 39319212 PMC11420642

[65] Ihle CL Wright-Hobart SJ Owens P. Therapeutics targeting the metastatic breast cancer bone microenvironment. Pharmacol Ther. (2022) 239:108280. doi: 10.1016/j.pharmthera.2022.108280, PMID: 36116682

[66] Lamouline A Bersini S Moretti M. *In vitro* models of breast cancer bone metastasis: analyzing drug resistance through the lens of the microenvironment. Front Oncol. (2023) 13:1135401. doi: 10.3389/fonc.2023.1135401, PMID: 37182144 PMC10168004

[67] Wu Q Tian P He D Jia Z He Y Luo W. SCUBE2 mediates bone metastasis of luminal breast cancer by modulating immune-suppressive osteoblastic niches. Cell Res. (2023) 33:464–78. doi: 10.1038/s41422-023-00810-6, PMID: 37142671 PMC10235122

[68] Khrystoforova I Shochat-Carvalho C Harari R Henke K Woronowicz K Harris MP. Zebrafish mutants reveal unexpected role of Lrp5 in osteoclast regulation. Front Endocrinol (Lausanne). (2022) 13:985304. doi: 10.3389/fendo.2022.985304, PMID: 36120446 PMC9478031

[69] Whitman MA Mantri M Spanos E Estroff LA De Vlaminck I Fischbach C. Bone mineral density affects tumor growth by shaping microenvironmental heterogeneity. Biomaterials. (2025) 315:122916. doi: 10.1016/j.biomaterials.2024.122916, PMID: 39490060 PMC11658005

[70] Kolahi Azar H Gharibshahian M Rostami M Mansouri V Sabouri L Beheshtizadeh N. The progressive trend of modeling and drug screening systems of breast cancer bone metastasis. J Biol Eng. (2024) 18:14. doi: 10.1186/s13036-024-00408-5, PMID: 38317174 PMC10845631

[71] Ravi P Ghosh S Pashaki PV Shetty K Kim J Gaba A. Evaluating breast cancer patient-specific metastasis severity at bone site using *in vitro* models. ACS Biomater Sci Eng. (2025) 11:2824–33. doi: 10.1021/acsbiomaterials.4c01599, PMID: 40168530

[72] Ravi P Jasuja H Sarkar D Vahidi Pashaki B Gaikwad HK Vahidi Pashaki P. Rhodiola crenulata induces apoptosis in bone metastatic breast cancer cells via activation of caspase-9 and downregulation of MtMP activity. Sci Rep. (2025) 15:9341. doi: 10.1038/s41598-025-93274-0, PMID: 40102501 PMC11920079

[73] Martínez-Pena I Hurtado P Carmona-Ule N Abuín C Dávila-Ibáñez AB Sánchez L. Dissecting breast cancer circulating tumor cells competence via modelling metastasis in zebrafish. Int J Mol Sci. (2021) 22:9279. doi: 10.3390/ijms22179279, PMID: 34502201 PMC8431683

[74] Zhang Y Alexander PB Wang XF. TGF-β Family signaling in the control of cell proliferation and survival. Cold Spring Harb Perspect Biol. (2017) 9:a022145. doi: 10.1101/cshperspect.a022145, PMID: 27920038 PMC5378054

[75] Derynck R Turley SJ Akhurst RJ. TGFβ biology in cancer progression and immunotherapy. Nat Rev Clin Oncol. (2021) 18:9–34. doi: 10.1038/s41571-020-0403-1, PMID: 32710082 PMC9721352

[76] Goumans MJ Ten Dijke P. TGF-β Signaling in control of cardiovascular function. Cold Spring Harb Perspect Biol. (2018) 10:a022210. doi: 10.1101/cshperspect.a022210, PMID: 28348036 PMC5793760

[77] Li C Ma J Groenewoud A Ren J Liu S Snaar-Jagalska BE. Establishment of embryonic zebrafish xenograft assays to investigate TGF-β Family signaling in human breast cancer progression. Methods Mol Biol. (2022) 2488:67–80. doi: 10.1007/978-1-0716-2277-3_6, PMID: 35347683

[78] Tulotta C Stefanescu C Beletkaia E Bussmann J Tarbashevich K Schmidt T. Inhibition of signaling between human CXCR4 and zebrafish ligands by the small molecule IT1t impairs the formation of triple-negative breast cancer early metastases in a zebrafish xenograft model. Dis Model Mech. (2016) 9:141–53. doi: 10.1242/dmm.023275, PMID: 26744352 PMC4770151

[79] Zhao C Wang X Zhao Y Li Z Lin S Wei Y. A novel xenograft model in zebrafish for high-resolution investigating dynamics of neovascularization in tumors. PLoS One. (2011) 6:e21768. doi: 10.1371/journal.pone.0021768, PMID: 21765912 PMC3135597

[80] Lee SH Jeong D Han YS Baek MJ. Pivotal role of vascular endothelial growth factor pathway in tumor angiogenesis. Ann Surg Treat Res. (2015) 89:1–8. doi: 10.4174/astr.2015.89.1.1, PMID: 26131438 PMC4481026

[81] Sennino B McDonald DM. Controlling escape from angiogenesis inhibitors. Nat Rev Cancer. (2012) 12:699–709. doi: 10.1038/nrc3366, PMID: 23001349 PMC3969886

[82] Ye W. The complexity of translating anti-angiogenesis therapy from basic science to the clinic. Dev Cell. (2016) 37:114–25. doi: 10.1016/j.devcel.2016.03.015, PMID: 27093081

[83] Okuda KS Misa JP Oehlers SH Hall CJ Ellett F Alasmari S. A zebrafish model of inflammatory lymphangiogenesis. Biol Open. (2015) 4:1270–80. doi: 10.1242/bio.013540, PMID: 26369931 PMC4610225

[84] Britto DD Wyroba B Chen W Lockwood RA Tran KB Shepherd PR. Macrophages enhance Vegfa-driven angiogenesis in an embryonic zebrafish tumor xenograft model. Dis Model Mech. (2018) 11:dmm035998. doi: 10.1242/dmm.035998, PMID: 30396905 PMC6307908

[85] Ghajar CM Peinado H Mori H Matei IR Evason KJ Brazier H. The perivascular niche regulates breast tumor dormancy. Nat Cell Biol. (2013) 15:807–17. doi: 10.1038/ncb2767, PMID: 23728425 PMC3826912

[86] Al-Hamaly MA Turner LT Rivera-Martinez A Rodriguez A Blackburn JS. Zebrafish cancer avatars: A translational platform for analyzing tumor heterogeneity and predicting patient outcomes. Int J Mol Sci. (2023) 24:2288. doi: 10.3390/ijms24032288, PMID: 36768609 PMC9916713

[87] Tomko A O’Leary L Trask H Achenbach JC Hall SR Goralski KB. Antitumor activity of abnormal cannabidiol and its analog O-1602 in taxol-resistant preclinical models of breast cancer. Front Pharmacol. (2019) 10:1124. doi: 10.3389/fphar.2019.01124, PMID: 31611800 PMC6777324

[88] Drabsch Y He S Zhang L Snaar-Jagalska BE ten Dijke P. Transforming growth factor-β signalling controls human breast cancer metastasis in a zebrafish xenograft model. Breast Cancer Res. (2013) 15:R106. doi: 10.1186/bcr3573, PMID: 24196484 PMC3978640

[89] Halder SK Beauchamp RD Datta PK. A specific inhibitor of TGF-beta receptor kinase, SB-431542, as a potent antitumor agent for human cancers. Neoplasia. (2005) 7:509–21. doi: 10.1593/neo.04640, PMID: 15967103 PMC1501161

[90] Inman GJ Nicolás FJ Callahan JF Harling JD Gaster LM Reith AD. SB-431542 is a potent and specific inhibitor of transforming growth factor-beta superfamily type I activin receptor-like kinase (ALK) receptors ALK4, ALK5, and ALK7. Mol Pharmacol. (2002) 62:65–74. doi: 10.1124/mol.62.1.65, PMID: 12065756

[91] Sliva D Rizzo MT English D. Phosphatidylinositol 3-kinase and NF-kappaB regulate motility of invasive MDA-MB-231 human breast cancer cells by the secretion of urokinase-type plasminogen activator. J Biol Chem. (2002) 277:3150–7. doi: 10.1074/jbc.M109579200, PMID: 11689575

[92] Sabeh F Shimizu-Hirota R Weiss SJ. Protease-dependent versus -independent cancer cell invasion programs: three-dimensional amoeboid movement revisited. J Cell Biol. (2009) 185:11–9. doi: 10.1083/jcb.200807195, PMID: 19332889 PMC2700505

[93] Zhong J Xiao C Chen Q Pan X Xu T Wang Y. Zebrafish functional xenograft vasculature platform identifies PF-502 as a durable vasculature normalization drug. iScience. (2023) 26:107734. doi: 10.1016/j.isci.2023.107734, PMID: 37680473 PMC10480778

[94] Hou W Xiao C Zhou R Yao X Chen Q Xu T. Inhibiting autophagy selectively prunes dysfunctional tumor vessels and optimizes the tumor immune microenvironment. Theranostics. (2025) 15:258–76. doi: 10.7150/thno.98285, PMID: 39744218 PMC11667230

[95] Wan YX Qi XW Lian YY Liu ZY Wang H Qiu YQ. Electroacupuncture facilitates vascular normalization by inhibiting Glyoxalase1 in endothelial cells to attenuate glycolysis and angiogenesis in triple-negative breast cancer. Cancer Lett. (2024) 598:217094. doi: 10.1016/j.canlet.2024.217094, PMID: 38945204

[96] Xun X Ai J Feng F Hong P Rai S Liu R. Adverse events of bevacizumab for triple negative breast cancer and HER-2 negative metastatic breast cancer: A meta-analysis. Front Pharmacol. (2023) 14:1108772. doi: 10.3389/fphar.2023.1108772, PMID: 36794276 PMC9922898

[97] Zhang J Gao B Zhang W Qian Z Xiang Y. Monitoring antiangiogenesis of bevacizumab in zebrafish. Drug Des Devel Ther. (2018) 12:2423–30. doi: 10.2147/DDDT.S166330, PMID: 30122900 PMC6084084

[98] Harfouche R Basu S Soni S Hentschel DM Mashelkar RA Sengupta S. Nanoparticle-mediated targeting of phosphatidylinositol-3-kinase signaling inhibits angiogenesis. Angiogenesis. (2009) 12:325–38. doi: 10.1007/s10456-009-9154-4, PMID: 19685150

[99] Zheng MW Zhang CH Chen K Huang M Li YP Lin WT. Preclinical evaluation of a novel orally available SRC/raf/VEGFR2 inhibitor, SKLB646, in the treatment of triple-negative breast cancer. Mol Cancer Ther. (2016) 15:366–78. doi: 10.1158/1535-7163.MCT-15-0501, PMID: 26721945

[100] Zou G Zhang X Wang L Li X Xie T Zhao J. Herb-sourced emodin inhibits angiogenesis of breast cancer by targeting VEGFA transcription. Theranostics. (2020) 10:6839–53. doi: 10.7150/thno.43622, PMID: 32550907 PMC7295066

[101] Wang D Liu X Hong W Xiao T Xu Y Fang X. Muscone abrogates breast cancer progression through tumor angiogenic suppression via VEGF/PI3K/Akt/MAPK signaling pathways. Cancer Cell Int. (2024) 24:214. doi: 10.1186/s12935-024-03401-6, PMID: 38898449 PMC11188526

[102] Yang H Jia H Zhao Q Luo KQ. Visualization of natural killer cell-mediated killing of cancer cells at single-cell resolution in live zebrafish. Biosens Bioelectron. (2022) 216:114616. doi: 10.1016/j.bios.2022.114616, PMID: 35963115

[103] Yang K Zhao Y Sun G Zhang X Cao J Shao M. Clinical application and prospect of immune checkpoint inhibitors for CAR-NK cell in tumor immunotherapy. Front Immunol. (2022) 13:1081546. doi: 10.3389/fimmu.2022.1081546, PMID: 36741400 PMC9892943

[104] Wang Y Li J Wang Z Liu Y Wang T Zhang M. Comparison of seven CD19 CAR designs in engineering NK cells for enhancing anti-tumor activity. Cell Prolif. (2024) 57:e13683. doi: 10.1111/cpr.13683, PMID: 38830795 PMC11533075

[105] Roy D Subramaniam B Chong WC Bornhorst M Packer RJ Nazarian J. Zebrafish-A suitable model for rapid translation of effective therapies for pediatric cancers. Cancers (Basel). (2024) 16:1361. doi: 10.3390/cancers16071361, PMID: 38611039 PMC11010887

[106] de Souza AM da Silva Junior FC Dantas ÉD Galvão-Pereira MC de Medeiros SRB Luchiari AC. Temperature effects on development and lifelong behavior in zebrafish. Sci Total Environ. (2025) 973:179172. doi: 10.1016/j.scitotenv.2025.179172, PMID: 40112540

[107] Dakappa PH Mahabala C. Analysis of long-term temperature variations in the human body. Crit Rev BioMed Eng. (2015) 43:385–99. doi: 10.1615/CritRevBiomedEng.2016016543, PMID: 27480582

[108] Cabezas-Sainz P Coppel C Pensado-López A Fernandez P Muinelo-Romay L López-López R. Morphological abnormalities and gene expression changes caused by high incubation temperatures in zebrafish xenografts with human cancer cells. Genes (Basel). (2021) 12:113. doi: 10.3390/genes12010113, PMID: 33477746 PMC7832305

[109] Lucianò AM Di Martile M Pérez-Oliva AB Di Caprio M Foddai ML Buglioni S. Exploring association of melanoma-specific Bcl-xL with tumor immune microenvironment. J Exp Clin Cancer Res. (2023) 42:178. doi: 10.1186/s13046-023-02735-9, PMID: 37488586 PMC10364435

[110] Ali Z Vildevall M Rodriguez GV Tandiono D Vamvakaris I Evangelou G. Zebrafish patient-derived xenograft models predict lymph node involvement and treatment outcome in non-small cell lung cancer. J Exp Clin Cancer Res. (2022) 41:58. doi: 10.1186/s13046-022-02280-x, PMID: 35139880 PMC8827197

[111] Zhan D Wang X Zheng Y Wang S Yang B Pan B. Integrative dissection of 5-hydroxytryptamine receptors-related signature in the prognosis and immune microenvironment of breast cancer. Front Oncol. (2023) 13:1147189. doi: 10.3389/fonc.2023.1147189, PMID: 37795441 PMC10546427

[112] Paul CD Bishop K Devine A Paine EL Staunton JR Thomas SM. Tissue architectural cues drive organ targeting of tumor cells in zebrafish. Cell Syst. (2019) 9:187–206.e116. doi: 10.1016/j.cels.2019.07.005, PMID: 31445892 PMC8276582

[113] Verma M Rhodes M Shinton S Wiest DL Simple A. Rapid, and effective method for tumor xenotransplantation analysis in transparent zebrafish embryos. J Vis Exp. (2024) 209. doi: 10.3791/66164, PMID: 39072643 PMC11370749

[114] Rodriguez-Tirado C Entenberg D Li J Qian BZ Condeelis JS Pollard JW. Interleukin 4 controls the pro-tumoral role of macrophages in mammary cancer pulmonary metastasis in mice. Cancers (Basel). (2022) 14:4336. doi: 10.3390/cancers14174336, PMID: 36077870 PMC9454655

[115] Winnard PT Jr. Vesuna F Bol GM Gabrielson KL Chenevix-Trench G Ter Hoeve ND. Targeting RNA helicase DDX3X with a small molecule inhibitor for breast cancer bone metastasis treatment. Cancer Lett. (2024) 604:217260. doi: 10.1016/j.canlet.2024.217260, PMID: 39306228

[116] Garone ME Chase SE Zhang C Krendel M. Myosin 1e deficiency affects migration of 4T1 breast cancer cells. Cytoskeleton (Hoboken). (2024) 81:723–36. doi: 10.1002/cm.21819, PMID: 38140937 PMC11193843

[117] Song F Yi X Zheng X Zhang Z Zhao L Shen Y. Zebrafish patient-derived xenograft system for predicting carboplatin resistance and metastasis of ovarian cancer. Drug Resist Update. (2025) 78:101162. doi: 10.1016/j.drup.2024.101162, PMID: 39571238

[118] Mendes RV Ribeiro JM Gouveia H Rebelo de Almeida C Castillo-Martin M Brito MJ. Zebrafish Avatar testing preclinical study predicts chemotherapy response in breast cancer. NPJ Precis Oncol. (2025) 9:94. doi: 10.1038/s41698-025-00882-0, PMID: 40169839 PMC11961725

[119] Weiss JM Lumaquin-Yin D Montal E Suresh S Leonhardt CS White RM. Shifting the focus of zebrafish toward a model of the tumor microenvironment. Elife. (2022) 11:e69703. doi: 10.7554/eLife.69703, PMID: 36538362 PMC9767465

[120] Saraiva SM Gutiérrez-Lovera C Martínez-Val J Lores S Bouzo BL Díez-Villares S. Edelfosine nanoemulsions inhibit tumor growth of triple negative breast cancer in zebrafish xenograft model. Sci Rep. (2021) 11:9873. doi: 10.1038/s41598-021-87968-4, PMID: 33972572 PMC8110995

[121] Larsson P Pettersson D Olsson M Sarathchandra S Abramsson A Zetterberg H. Repurposing proteasome inhibitors for improved treatment of triple-negative breast cancer. Cell Death Discov. (2024) 10:57. doi: 10.1038/s41420-024-01819-5, PMID: 38286854 PMC10825133

[122] Noureddine LM Ablain J Surmieliova-Garnès A Jacquemetton J Pham TH Marangoni E. PRMT5 triggers glucocorticoid-induced cell migration in triple-negative breast cancer. Life Sci Alliance. (2023) 6:e202302009. doi: 10.26508/lsa.202302009, PMID: 37536978 PMC10400884

[123] Guo Y Fan Y Pei X. Fangjihuangqi Decoction inhibits MDA-MB-231 cell invasion *in vitro* and decreases tumor growth and metastasis in triple-negative breast cancer xenografts tumor zebrafish model. Cancer Med. (2020) 9:2564–78. doi: 10.1002/cam4.2894, PMID: 32037729 PMC7131862

[124] Shiau JP Wu CC Chang SJ Pan MR Liu W Ou-Yang F. FAK regulates VEGFR2 expression and promotes angiogenesis in triple-negative breast cancer. Biomedicines. (2021) 9:1789. doi: 10.3390/biomedicines9121789, PMID: 34944605 PMC8698860

